# Morphological and Physiological Research on Fish

**DOI:** 10.3390/ani15111513

**Published:** 2025-05-22

**Authors:** Paola Scocco, Elena De Felice

**Affiliations:** School of Biosciences and Veterinary Medicine, University of Camerino (MC), 62032 Camerino, Italy; paola.scocco@unicam.it

The Special Issue “Morphological and Physiological Research on Fish” is dedicated to the application of morphological and physiological studies carried out on fish, attempting to provide information regarding the relevance of morphological studies and their relationships to functions. Indeed, fish not only represent the largest and most varied group of vertebrates but there are also numerous applications for these animals in both research and industry. Moreover, aquaculture is one of the world’s most efficient and sustainable methods of producing high-quality protein and, in recent decades, fish have emerged as an interesting model system in biomedical research, due to the close similarities they have with mammals in terms of various basic mechanisms. Morphological studies provide us with context for the comprehension of the spatial organization and relationship between physiological and biochemical data, and the molecular machinery that is promptly being explained through molecular techniques directed at the genome, transcriptome, and proteome. As a result, new morphological and physiological investigations of fish would enhance our understanding of these creatures, with exceptional and interesting relevance both in basic and applied research.

Many authors contributed to the Special Issue with ten original articles and four reviews, and the range of topics covered extended from descriptive morphology to morphometry, from stress markers to toxic agents, from wild to aquaculture or model species, involving different organs and apparatus. Morphometrical and morphological studies were the most numerous contributions to this Special Issue.

*Hemibarbus maculatus*, a small freshwater fish of the family Cyprinidae, is a common economic fish in the midstream and downstream of the Jialing River, China). In order to resolve the difficulties in aquacultural cultivation of this fish (H. maculatus usually die quickly and do not grow to the same size as the wild ones)—Wu and colleagues [Contribution 1] tested the intestinal and liver digestive function of wild and cultured fishes. Histological staining and biochemical methods were used to compare the differences in morphological structure, goblet cells, argyrophil cells, lymphocytes, and Na+/K+ATPase in the intestine through hematoxylin eosin (H/E), periodic acid–Schiff in combination with the Alcian blue (AB-PAS) and silver leaching methods. To determine the gut absorption function, the Na+/K+-ATP enzyme’s vitality was detected. For the observation of liver tissue, H/E, PAS, and oil red O staining were used for morphology, glycogen, and lipid observations. The findings revealed that cultured *Hemibarbus maculatus* had a higher fat content attached to the gut, lower Na+/K+ATPase activity in both the foregut and hindgut (*p* < 0.01), and a reduced number of goblet cells in the hindgut (*p* < 0.01) compared to their wild counterparts. The number of argyrophilic cells did not differ significantly between the two groups, but the number of lymphocytes was notably lower in the gut segments of the cultured fish. This indicates that the absorptive function and intestinal immunity are weaker in the cultured fishes. Additionally, the liver of cultured fish contained more glycogen and lipid, suggesting a decreased digestive function. In summary, the wild *Hemibarbus maculatus* exhibit better intestinal digestion, absorption, and lymphocyte levels than the cultured ones, which also show more hepatic lipopexia and glycogen accumulation. Future aquaculture practices should take these changes into account when addressing practical issues.

Among the most-used fish species in aquaculture is the Nile tilapia (*Oreochromis niloticus*), due to its rapid growth rate and its adaptation to a wide range of farming conditions. The aim of the work of Palladino and colleagues [Contribution 2] was an integrated description of esophagus and stomach morphology, crucial to gaining a deeper understanding of the various facets of this species’ physiological development and health. Combining scanning electron microscopy and morphometry, the thickness of the superficial and glandular epithelium, the submucosa, and the circular and longitudinal musculature of the stomach were measured. The authors highlight the presence of five different zones in the stomach (1: the esophagus–gastric lumen passage; 2: the descending glandular portion; 3: the fundic portion; 4: the ascending glandular portion; and 5: the gastric–pyloric transition portion). Histochemical investigation, consisting of AB/PAS and AB stainings at different pH levels, showed the secretion of carboxylates glycoconjugates along the esophagus and sulphated complex carbohydrates in the stomach. This dataset could be helpful for research aimed at enhancing animal welfare and production rates by better understanding the morphological alterations brought about by various feed regimens.

Beluga (*Huso huso*) and sevruga (*Acipenser stellatus*) are among the most important species of sturgeon fish (Acipenseridae) inhabiting the Caspian Sea, with demand for their products, such as caviar, meat, skin, and cartilage, being particularly high. The creation of specially designed compound diets to support growth and product quality is a crucial component of the sturgeon farming sector that influences both production efficiency and the sector’s long-term viability. However, the presence of digestive enzymes in various areas of the gastrointestinal tract is primarily responsible for the fish’s capacity to digest complicated meals. Trypsin, as an alkaline protease, is able to hydrolyze protein residues and peptides to release free amino acids and small peptides for intestinal absorption; therefore, the activity of trypsin has been widely used as a valuable indicator of digestive capacity in fish. In their work, Zamani and coworkers [Contribution 3] aimed to characterize trypsin from beluga and sevruga for the first time. The study’s findings indicate that the physicochemical and biochemical characteristics of trypsin from beluga and sevruga are consistent with information found in bony fish, and they could be used as a starting point for the development of in vitro tests to evaluate the digestibility of proteins in these archaic species.

The marbled flounder (*Pseudopleuronectes yokohamae*), which is distributed throughout Korea, southern Hokkaido in Japan, and the East China Sea, is a fish species with significant commercial value in both Korea and Japan, and it is gaining popularity as a new aquaculture target. The study by Cho and colleagues [Contribution 4] looked at the morphology, histology, and histochemistry of the digestive tract of *P. yokohamae*. The relative length of the gut (RLG) was measured, and samples from nine different regions of the digestive tract (the esophagus, cardiac stomach portion, fundic stomach portion, pyloric stomach portion, pyloric caeca, anterior intestine portion, mid intestine portion, posterior intestine portion, and rectum) were stained with Hansen’s hematoxylin and 0.5% eosin (H/E) for histological observation, in particular targeting the length of the mucosal folds and the thickness of the muscularis externa, which were stained with Alcian blue/periodic acid–Schiff (AB/PAS) for observations of mucus-secreting goblet cells. In this study, marbled flounder had an RLG value of 1.54 ± 0.10, like omnivore fish. However, the digestive tract’s form and structure resembled carnivorous fish. It possessed a simple stomach with six to nine pyloric caeca, and the mucosal folds of the digestive system had a generally branching architecture. Goblet cells were most abundant in the esophagus, followed by the anus, and CCK-producing cells, analyzed through immunohistochemistry, were detected at varying rates in the pyloric caeca and intestine, but not in the esophagus or the stomach. CCK-producing cells and goblet cells in the marbled flounder are effectively adapted to facilitate the ideal regulation of digestion. This work provides fundamental insights for further physiological and nutritional research.

The European sea bass, *Dicentrarchus labrax*, has great economic importance because it is one of the most important marine aquaculture fish species in the Mediterranean. Despite its widespread cultivation, this fish exhibits higher cortisol responses to stress than other commonly grown species in the Mediterranean. The purpose of the review by Samaras [Contribution 5] was to examine all previously published data on baseline and post-acute stress cortisol levels in this species. For this systematic review and meta-analysis, the Web of Science and Scopus databases were searched for studies reporting plasma or serum cortisol levels with no language or date constraints. The data were extracted directly for the reported results and were analyzed separately for basal and post-acute stress levels, as well as their standardized mean differences (SMDs) using random-effects meta-analyses. The current meta-analysis examined 69 studies. This meta-analysis concluded that there is significant variation between studies in both basal and post-stress concentrations. The test type seems to be one of the most important factors influencing heterogeneity. Studies employing ELISA assays had a considerably larger pooled effect size than studies using RIA assays at both the baseline and post-stress levels. As a result, it was impossible to definitively determine which assay type—ELISA or RIA—was more reliable in terms of reporting cortisol levels, but the current study supports the idea that the cortisol assay type should be carefully considered when organizing a study and interpreting the findings. Anesthesia also influenced cortisol levels, which caused the results to vary.

The Atlantic salmon business is predicted to expand internationally by 2–3% by 2030. The primary protein source in salmon diets is fishmeal (FM) (anchovies, pilchards, mackerel, herring, and blue whiting), which is derived from wild-caught marine fish whose natural stocks are in danger of depletion. It is therefore of fundamental importance to pick alternative feed ingredients that present a sufficient protein profile, guarantee a good health status and growth performance of Atlantic salmon, and are also environmentally sustainable. However, it is crucial to note that replacing FM with terrestrial plant elements may produce alterations and have a negative impact on the gastrointestinal tract. The utilization of plant components is limited in carnivorous fish species due to the presence of starch and structural carbohydrates, and a wide range of anti-nutritional factors. Diet can also have an impact on microbiota status, and many different plant-based proteins have been studied for their deleterious effects on the intestine. These changes may impair the animal’s digestive functions, development, and, eventually, well-being. Aidos and colleagues [Contribution 6] provide an updated review of alternative protein sources, including their impact on intestinal health in terms of morphology and microbial composition. They reviewed the literature on soybean meal, peas, faba beans, wheat, maize meal, sunflower meal, rapeseed meal, and wheat gluten. They also present animal protein sources that may serve as viable alternatives. A good example was the poultry by-product meal (from poultry processing); also black soldier fly and yellow meal worm. Such information may serve as a foundation for the selection and development of Atlantic salmon feeds that ensure fish health and growth performance while having a minimal impact on the surrounding environment, both in terms of the depletion of the fish’s natural stocks and pressure on terrestrial agriculture.

The review by De Marco and coworkers [Contribution 7] focuses on how feed additives containing prebiotics and probiotics affect fish microbiota. The number of microorganisms in a fish’s gastrointestinal tract (GIT) has been estimated to be between 107 and 108 per gram. However, the GIT microbiota is highly changeable, and the typical variation in quantity and variety of microorganisms is influenced by different factors, including age, nutrition, host genetics, and the environment (freshwater or oceanwater). Different sections of the fish GIT have been identified, each with a distinct microbial density. Indeed, a certain number of bacteria can be detected throughout the gastrointestinal tract, but some areas are more colonized than others. Aside from being identified as a very good alternative to the use of antibiotics, the use of these feed additives in aquaculture represents a useful strategy in terms of improving the overall performance of the aquaculture sector because it affects a variety of factors including rearing water quality, food absorption, and digestion, and has been shown to have a positive effect on the growth performance of fish. Furthermore, the use of histological assays as a valid technique for assessing fish welfare was highlighted, as were insights into nutritional absorption capacity and pathogen reactivity in fish using gut morphological endpoints. In particular, in histological studies of the fish intestine, endpoints such as the length of the mucosal folds (villi), muscle thickness, and crypt depth can provide useful information on intestinal efficiency in terms of nutrient absorption. Meanwhile, assessing the number of goblet cells and leukocytes could help to evaluate the state of the immune system response, which is very important for the balance of the gut microbiota. The evaluation of these endpoints can be performed using both light and electron microscopy and provides useful information to accurately evaluate the effects of prebiotics and probiotics at the level of the fish gastrointestinal tissue. Overall, gaining a solid knowledge base on the appropriate dosing of feed additives for individual fish species is a promising strategy to significantly improve the aquaculture sector in terms of both quality and sustainable production.

In aquaculture, transportation is a crucial process for transferring juveniles from the hatchery to the commercial farm, where they are reared before being sent to the food market. Furthermore, in farms located far from the slaughterhouse, transportation becomes essential to convey the fish to the facility where they are sacrificed and prepared for sale. Transportation can be stressful for fish due to handling, air exposure, confinement, and reduced oxygen levels. Therefore, it is crucial to gain insights into optimal practices for organizing transportation, ensuring the well-being of these animals and minimizing excessive stress. Bortoletti and colleagues [Contribution 8] investigated the effects of 24 h transport on the stress response in meager (*Argyrosomus regius*) juveniles by using radioimmunoassay to measure muscle cortisol levels and immunohistochemistry in order to detect the presence and intracellular localization of heat shock protein 70 (HSP70), 4-hydroxy-2-nonenal (HNE), 3-nitrotyrosine (NT), and 8-hydroxy-20-guanosine (8-OHdG) in various organs and tissues. The muscle cortisol levels in fish transferred to the transport tanks were significantly higher than those of the controls. Regarding immunohistochemistry, positive results for the various markers were detected in the epidermis of the skin, intestine, and gill lamellae, as well as the gastric glands and hepatopancreas. However, no substantial variations in signal intensity were observed when comparing control and transported animals, except in the case of HNE. The observed stress response was mostly related to loading stress and the transport process, highlighting the significance of developing proper operating procedures to protect fish well-being during transport. The unaltered distribution of oxidative stress indicators across the control and transferred groups showed that the stress experienced may be within acceptable limits.

Cytoplasmic linker-associated protein-2 (CLASP2) is a member of the CLIP-associating proteins (CLASPs) family and is involved in the structure and function of microtubules and Golgi apparatus; however, there are no studies that have clearly defined its role during fish germ cell formation. Ricci and coworkers [Contribution 9] aimed to analyze clasp2 gene transcription levels during fish germ line cell formation in zebrafish (*Danio rerio*) and guppy (*Poecilia reticulata*). Using real-time q-PCR, the authors found that clasp2 is transcribed in several tissues, such as brain, eye, and kidney tissues, in a comparable fashion between zebrafish and guppy; however, higher transcription was observed in the testis. By in situ hybridization in zebrafish, the authors found that clasp2 is specifically transcribed in the spermatozoa of adult testes. Interestingly, in the guppy, clasp2 is highly transcribed in spermatozeugmata. This study represents the first evidence of clasp2 expression in developing male germ line cells, providing insight that enables further investigation of this protein as a target in teleost fish meiotic cell division.

Bisphenol A (BPA) is a significant component utilized in the manufacture of technical plastic items. BPA is of relevance because of its possible detrimental effects on humans, as well as the fact that it is a prevalent pollutant in aquatic environments. The aim of the study by Smorodinskaya and colleagues [Contribution 10] was to determine the effects of different lethal and sublethal concentrations of BPA on zebrafish (*Danio rerio*) survival in an acute experiment investigating hematological parameters and hematopoiesis processes, as well as histological changes in the head kidney. The obtained data made it possible to determine the acute toxic limits of BPA for adult zebrafish. Concentrations exceeding 4 mg/L reliably resulted in fish mortality within 96 h, with an LC50 of 6.22 mg/L. Significant increases in the frequency of erythrocyte nuclear abnormalities were observed at BPA concentrations of 6 and 8 mg/L, which indicates a genotoxic effect. BPA’s impact on fish peripheral blood parameters manifested as an increase in the number of erythrocytes (RBC) and immature erythrocytes, as well as a decrease in the number of lymphocytes. The most notable pathological changes in the head kidney’s hematopoietic tissue included circulatory disturbances and the formation of inflammation/degradation foci. The conducted study makes it possible to conclude that BPA, at concentrations of 6 and 8 mg/L, influences the differentiation of cell elements in the erythroid series. Despite major advances in the study of hematopoiesis in lower vertebrates, including fish, morphological and quantitative assessment approaches still require refining. The authors of this work proposed a method for counting hematopoietic progenitor cells, focusing on elements from the erythropoietic and myelopoietic lineages. This method has potential uses in determining the impact of hazardous chemicals on hematopoiesis.

The poor quality of biological information, such as the relationship between length and weight parameters, could be a source of variability with a significant impact on stock assessment results. This relationship between the length and weight of individuals could differ between males and females as well as two separated islands and/or may be linked to the reproduction period of a species. Therefore, to estimate the morphometric relationships between total length and weight (the length–weight relationship, LWR) of each fish species according to potential spatial, temporal, and sex differences, Mahè and coworkers sampled 109 fish species (24,996 individuals) around the islands of Guadeloupe and Martinique from October 2021 to September 2022 [Contribution 11]. This is the first time that the LWR was estimated in the Atlantic Ocean for 16 species. There was a significant relationship between length and weight for all of the tested species. The sex effect on the LWR showed significant sexual dimorphism for 24 species. Additionally, a link between the temporal effect and the reproduction period was tested for 68 species, of which 35 presented significant differences relative to the annual quarter of sampling. Finally, the geographical effect (i.e., the difference between samples from around Guadeloupe and those from Martinique) was significant for 60 species. This island effect was significant for 25 species. These data and results will be essential for the stock assessment of Caribbean fishes.

Cadmium is one of the most toxic heavy metal contaminants in the environment and it can enter aquatic ecosystems via industrial waste. The use of chitosan to mitigate the negative effects of heavy metal contamination on aquaculture organisms is a common treatment strategy for eliminating heavy metal pollution in water. The aim of study by Zhang and colleagues [Contribution 12] was to investigate the effects of dietary chitosan supplementation on the muscle composition, digestion, lipid metabolism, and stress resistance, and their related gene expression, of genetically improved farmed (GIFT) juvenile tilapia (*Oreochromis niloticus*) subjected to cadmium stress. Compared to the control group, dietary supplementation with chitosan significantly increased the contents of crude protein and crude fat in the muscles and the activities of lipase, trypsin, and amylase in the intestines of juvenile GIFT exposed to cadmium-induced stress. These findings suggested that dietary supplementation with chitosan exhibited the potential to mitigate the adverse effects of cadmium-induced stress in terms of intestinal enzyme activity and improving digestive performance. In addition, dietary supplementation with chitosan could significantly upregulate the relative expression levels of hormone-sensitive lipase (hsl), peroxisome proliferator-activated receptor alpha (ppar*a*), lipoprotein lipase (lpl), malate dehydrogenase (mdh), carnitine palmitoyltransferase 1 (cpt-1), leptin (lep), fatty acid synthase (fas), stearoyl-CoA desaturase (scd), peroxisome proliferator-activated receptor gamma (ppar*γ*), and sterol regulatory element-binding protein 1 (srebp1) genes in the livers of GIFT juveniles exposed to cadmium-induced stress, suggesting that dietary supplementation with chitosan could effectively alleviate lipid metabolism impairment while concurrently augmenting their lipid metabolism capacity. These findings may provide scientific insights into the use of chitosan in aquaculture operations and the management of heavy metal contamination in aquatic ecosystems.

As the Mediterranean Sea continues to undergo transformations, the study of teleost reproduction serves as a critical lens through which to comprehend the broader implications of human activities on marine ecosystems and work towards their preservation. Lombò and coworkers [Contribution 13] in their review analyzed three stressors, namely climate change, environmental pollution, and overfishing, which influence various levels of the reproductive process in this changing scenario.

To better understand the population structure and biological background of fishery *Conger myriaster*, an economically important marine fish, five meristic counts and seventeen morphological measurements from seven different geographical populations in coastal China were analyzed. The traditional morphometry method was applied to analyze the standardized measurements together with the meristic counts so as to provide supplementary information for the fishery biology, population assessment, and fishery resource protection of this fish. The results of the work of Xiao and Yang [Contribution 14] showed that the greatest divergence was observed between the Dalian and Qingdao populations, whereas the smallest difference was found between the Lianyungang and Zhoushan populations. Statistical differences in tail length (*TAL*) were detected between all populations. The morphological traits with high variation coefficient values were mostly related to body weight (*BW*), confirming greater potential variations in these weight-related traits. Principal component analysis (PCA) extracted seven principal components (PCs) with eigenvalues greater than 1, and the cumulative contribution rate was 72.790%. The results of cluster analysis, together with the PCA and DFAs (discriminant function analyses), supported separating the populations into three groups linked with their geographic distribution and their specific environment localization. Considering the particularity of the natural environment of the Bohai Sea and the sophisticated oceanic circulations of the Shandong Peninsula, the relationships of *C. myriaster* populations in the northwest Pacific Ocean along the China coast were closely related to their geographical distributions and oceanic circulations. Understanding the composition of a population in terms of its demographic characteristics, spatial distribution, and social organization is of great importance for policymakers, researchers, and organizations, enabling them to make informed decisions and develop effective strategies.

In conclusion, we can state that there is a very broad interest in the fish world, ranging from basic to applied research. Studies on fish morphology and physiology are critical to help us understand their adaptation, ecological roles, and evolutionary relevance. They are critical to conservation efforts, sustainable aquaculture, and fisheries management, guaranteeing biodiversity and encouraging responsible resource use. Exploring these elements provides scientists and researchers with significant insights into the resilience and functionality of aquatic life, which benefits both ecosystems and human enterprises. We believe that this Special Issue may be of interest, and we hope that this topic will be increasingly explored in the future.

